# Ecophysiological Parameters of Medicinal Plant *Filipendula vulgaris* in Diverse Habitat Conditions

**DOI:** 10.3390/biology11081198

**Published:** 2022-08-10

**Authors:** Beata Barabasz-Krasny, Katarzyna Możdżeń, Agnieszka Tatoj, Katarzyna Rożek, Peiman Zandi, Ewald Schnug, Alina Stachurska-Swakoń

**Affiliations:** 1 Department of Botany, Institute of Biology, Pedagogical University of Krakow, 30-084 Cracow, Poland; 2 Independent Research, 30-046 Krakow, Poland; 3 Institute of Botany, Jagiellonian University, 30-387 Cracow, Poland; 4 International Faculty of Applied Technology, Yibin University, Yibin 644000, China; 5 Department of Life Sciences, Institute for Plant Biology, Technical University of Braunschweig, 38-106 Braunschweig, Germany

**Keywords:** PSII activity, biomass, chlorophyll, electrolyte outflow, arbuscular mycorrhiza, *Molinia* meadow, xerothermic grassland

## Abstract

**Simple Summary:**

Dropwort (*Filipendula vulgaris* Moench) is a perennial plant (hemicryptophyte), growing on xerothermic grasslands *Festuco-Brometea* and changing-wet *Molinia* meadows in the Eurasian area. Due to the production of active substances, the species is used in folk medicine and phytotherapy. This study includes determining which of the two different habitats occupied by *F. vulgaris* creates better conditions for its growth and development. Selected physiological parameters of dropwort plants (PSII activity, chlorophyll content, electrolyte leakage, hydrogen peroxide content, and biomass), the occurrence of mycorrhiza, and soil characteristics were investigated. Soil analysis showed a higher content of nutrients in grasslands, and a higher content of heavy metals in meadows. Plants of *F. vulgaris* growing in the wet meadows achieved a significantly lower mass compared to plants growing in grasslands. The colonization degree of *F. vulgaris* by arbuscular mycorrhizal fungi (AMF) from both stands oscillated around high values; dropwort formed the *Arum* type of mycorrhiza. A much higher content of chlorophylls was observed in plants from grasslands. *F. vulgaris* showed different photosynthetic activity depending on the habitat. Based on chlorophyll fluorescence imaging, higher activity was found in plants from grasslands, compared to plants from meadows, but in specimens from grasslands, there are symptoms of damage to the PSII system. The analyses carried out showed that better conditions for growth and physiological activity of this species are probably associated with grasslands on a calcareous substrate, although the irradiance stress of excess light is visible, manifested, e.g., by little dysfunction of photosynthetic structures.

**Abstract:**

This study attempts to determine which of the habitats occupied by *Filipendula vulgaris* creates better conditions for its growth and development. Selected physiological parameters—PSII activity, chlorophyll content, electrolyte leakage, hydrogen peroxide content as well as biomass, the occurrence of mycorrhiza, and soil characteristics—were investigated. Grassland soils had a higher content of macronutrients and a lower concentration of heavy metals. The degree of colonization of *F. vulgaris* by AMF (*Arum* type) oscillated around high values in both types of stands. Plants growing on xerothermic grasslands achieved much better fluorescence parameters than those collected from meadows. Similar results were obtained from the analysis of chlorophyll content. The destabilization degree of cell membranes was significantly higher in plants collected in meadows than in grasslands. Biomass analysis showed higher values of these parameters in grassland plants. In the case of the parameters of fluorescence emission, plants growing on grasslands achieved significantly lower values than plants collected from meadows. The analyses carried out showed that better conditions for growth and physiological activity of *F. vulgaris* are probably associated with grasslands on a calcareous substrate.

## 1. Introduction

The photosynthesis process is very sensitive to various factors of abiotic stress, e.g., [1,2,3,4]. Its correct course is a good indicator of the physiological condition of plants. The intensity of photosynthetically active solar radiation is a parameter that directly or indirectly affects photosynthetic photochemistry [3,5]. For example, a symptom of too high level of solar radiation may be the degradation of chlorophylls necessary for photosynthesis. Therefore, plants adapt to light through structural adaptations and plant pigments, e.g., [6,7,8]. For plants grown in extremely shaded conditions, the light of several thousand lux can inhibit photosynthesis, while in other plants this effect only occurs at levels above 100,000 lux. In the natural environment, the spectral composition and light intensity change significantly during the growing season, especially during the growth and development of leaves [4,9]. The sensitivity of plants to light and other environmental factors is thus the result of phylo- and ontogenetic adaptation.

Plant organisms have built up physical and endogenous barriers to absorb or dis-perse excess solar radiation, e.g., [10,11,12]. This adaptation is crucial for their survival in conditions of excess light availability, which excess may also damage the photosynthesis structures. An interesting adaptation is the so-called phenotypic plasticity, due to which the same set of genes can create different phenotypes of plants when exposed to various environmental factors, such as light availability, temperature, soil conditions, or water availability. Thus, phenotypic plasticity concerns individuals experiencing various environmental conditions, in which developmental instability is the result of random variability of developmental processes, causing deviations in each developing structure from the norm expected for this genotype and environment [13]. This phenomenon is assigned a very important role in the adaptation of plants to the changing conditions of the natural environment [14,15,16,17].

The plants displaying plasticity in terms of the occurrence in habitats with extreme water conditions include dropwort (*Filipendula vulgaris* Moench; syn. *F. hexapetala* Gilib, Rosaceae Juss.), which grows in dry and wet soils. The species is a perennial with a straight flower stalk up to 80 cm long. It has double-odd-pinnate leaves, clustered in a rosette at the base of the shoot. Its flowers are small, cream colored, gathered in a paniculate inflorescence, pollinated by insects or wind [18,19]. It produces short, tuberous rhizomes with clonal growth [20]. It belongs to the Eurosiberian species [21]; occurs in northwestern Africa, Europe, and Central Asia, up to Siberia. It is a diagnostic taxon of the *Festuco-Brometea* Br.-Bl. et R.Tx. 1943 class, which includes calcareous thermophilic grasslands [22,23,24]. Moreover, this species can also grow on wet meadows of the order Molinietalia caeruleae W. Koch 1926 and rarely in the Central Europe low peat bogs *Caricetalia davalianae* Br.-Bl. 1949 [25,26], both on calcareous deposits. So far, studies focusing on its biology and habitat interactions are scarce [19,27]. Most researchers were interested in the medicinal properties of this plant. This species has long been used for medicinal purposes in countries such as Poland [28,29], Romania [30], Russia [31], and Serbia [32]. As a result of its population disappearing in Europe, it has been included in many local *Red Lists*, as well as the European *Red List of Medicinal Plants*.

The aim of the research was the estimation of the physiological activity of the dropwort (*Filipendula vulgaris* Moench) of two semi-natural habitats extreme in soil moisture (wet meadows and xerothermic grasslands). Therefore, attempts were made to find answers to the following questions: (1) Do the occurrence stands of *F. vulgaris* differ in the content of macroelements and heavy metals in the soil? (2) Do the plants growing in the *Molinia* meadows differ from those from xerothermic grasslands in terms of biomass and mycorrhiza? (3) Does the type of stand (habitat) affect the chlorophyll content in dropwort leaves? (4) Does dropwort, depending on the stand, exhibit different photosynthetic activity? (5) Which habitat conditions are more stressful for the dropwort: water stress or light stress?

## 2. Materials and Methods

### 2.1. Characteristics of the Study Area and Plants Material

Specimens of *Filipendula vulgaris* were collected in July 2019 from two semi-natural habitats in southern Poland: wet *Molinia* meadows (50°01′48.8″ N 19°52′26.7″ E) and calcareous xerothermic grasslands (50°18′59.1″ N 20°04′14.3″ E). According to the Meteorological Yearbook, the temperature was typical for July and this region of Poland—approximately 19 °C; rainfall was 74.7 mm, slightly below the July average—107 mm. These specific sites were selected due to the varied habitat conditions, expressed also in the floristic composition, and the appropriate population size of *F. vulgaris*, as the species is rare in its northern range.

(MM) Wet meadows of the *Molinion* W. Koch 1926 alliance (the *Molinio-Arrhenatheteretea* R.Tx. 1937 class) were located on the floodplain terrace of the Vistula River, with limestone outliers (Jurassic-Cretaceous) and tectonic depression of the Brama Krakowska gate [27]. The Areni-Humic Gleysols (hydrogenous soil) are listed as the soil unit for this site [33]. The hydrological conditions vary throughout the growing season—in spring and early summer, the groundwater level is high, and could be above the soil surface, and in August it drops to the level typical for fresh meadows—below the soil surface. Species such as *Betonica officinalis*, *Inula salicina*, *Lythrum salicaria*, *Molinia caerulea*, *Sanquisorba officinalis* occur in the area. As the mowing is not regularly provided, the succession process with *Phragmites australis* expanding is observed.

(XG) Xerothermic grasslands were represented by the *Thalictro-Salvietum* Medw.-Kornaś 1959 association from the *Festuco-Brometea* Br.-Bl. et R.Tx. 1943 class, were located in the Miechów Upland. The rendzinas and limestone leptosols are the dominant soil units in this area. There is a constant water deficit here, only after the spring thaw the humidity conditions are good here. Between species plants growing here: *Anthericum ramosum*, *Brachypodium pinnatum*, *Centaurea scabiosa*, *Elymus hispidus*, *Salvia pratensis*, *S. verticillata*, and others. Such phytocoenoses are usually extensively grazed. Cessation of grazing provides for succession into forest-shrub communities.

### 2.2. Soil Analysis

To determine the content of selected macroelements and heavy metals on the stands of *F. vulgaris*, mixed soil samples were taken from two habitats: a variable-wet *Molinia* meadow with hydrogenic soil and a xerothermic grassland with calcareous rendzina. From the top layer of the soil profile (5–20 cm), the soil samples were collected at 10 points and then mixed to obtain a general sample. Five general samples were taken for each stand. Soil samples were dried and sieved with 2 mm sieve. For both stands, soil reaction (pH in KCl) and assimilable forms of macro- and microelements were examined using the Mehlich-3 method [34], by means of inductively coupled plasma optical emission spectrometry with Avio 200 ICP Optical Emission Spectrometer (PerkinElmer Inc.). This method is based on calorimetry and flame photometry of the content of elements [35]. It is the standard method used in agricultural soil monitoring, it allows to test in one soil extract the content of basic macronutrients, such as P, K, and Mg, but also other important nutrients-S, Ca or micronutrients, e.g., B and Cu [36]. NO_3_-N was extracted by shaking in H_2_O (in 1:5, *w*:*v*) and measured with Laquatwin Nitrate Ion meter (Horiba co.) according to factory protocol.

### 2.3. Root Staining and the Examination of Fungal Root Colonization

In order to determine the presence of fungal endophytes, the roots of *F. vulgaris* growing on xerothermic grassland, *Molinia* meadow, and succession stages were collected (5 individuals per habitat). Roots of dropwort were stained by Philips and Hayman [37] method, with modifications [38]. From each sample, a ~1 cm-long 30 randomly selected fragments of fine roots were mounted with glycerol: lactic acid (1:1, *v*:*v*) on a microscope slide and pressed by a coverslip. The morphology of AM was evaluated following Dickson [39]. Colonization of AMF and the presence of endophytes were calculated following Trouvelot et al. [40] method by using Nikon Eclipse 80i light microscope with differential interference contrast (DIC). The following parameters of AMF colonization degree were calculated: mycorrhizal frequency F(%)—the ratio between roots colonized by AMF and the total number of root fragments, relative mycorrhizal root length M(%)—the proportion of root cortex colonized by AMF relative to the total root system, and relative arbuscular richness A(%)—arbuscule abundance in the whole root system [40]. For DSE presence, parameter of frequency F(%) was calculated [41].

### 2.4. Chlorophyll a Fluorescence

Chlorophyll *a* fluorescence imaging from *F. vulgaris* leaves (from 5 specimens per habitat) was performed in a closed FluorCam FC 800C measuring chamber (Photon Systems Instruments, Drásov, Czech Republic) [42,43]. To quench the light photosynthesis phase, the leaves were placed on filter paper soaked in distilled water and allowed to darken for 30 min. After this time, the leaves were exposed to light and selected fluorescence parameters were determined: F_0_—zero fluorescence, F_m_—maximum fluorescence, F_v_/F_m_—maximum photochemical efficiency of PSII, NPQ—non-photochemical quenching, and Rfd—PSII vitality indicator. In each case, the source of the red color is chlorophyll particles and the PSII antenna system from chloroplasts of mesophilic cells [44].

### 2.5. Chlorophyll Content

The content of chlorophyll *a* and *b* was determined by the spectrophotometric method according to Barnes et al. [45]. Discs with a diameter of 1 cm were cut by cork-borer from the tested leaves of *F. vulgaris* (from 5 specimens per habitat), which were then weighed on a laboratory balance with an accuracy of 0.0001 g (Ohaus Adventurer Pro, Parsippany, NJ, USA) and extracted in 3 mL of dimethyl- sulfoxide (SIGMA-Aldrich, St. Louis, MO, USA) for 48 h at 65 °C. The chlorophyll extract was poured into 1 mL polypropylene cuvettes and measured on a spectrophotometer Aquarius 9500 (Cecil Instruments, Cambridge, UK), at two wavelengths λ = 648 and 665 nm.

Chl *a* = [(14.85 × A665 − 5.14 × A648) × V]/(1000 × W)

Chl *b* = [(25.48 × A648 − 7.36 × A665) × V]/(1000 × W)

Sum *a* + *b* = [(7.49 × A665 + 20.34 × A648) × V]/(1000 × W)

Ratio *a*/*b* = Chl *a*/Chl *b*

Chl—chlorophyll, A—absorbance at a given wavelength, V—total volume of the extract (mL), and W—sample mass (g).

### 2.6. Chlorophyll Fluorescence Emission

The blue-green and red fluorescence emission spectra were measured on a spectrofluorometer LS-55B (PerkinElmer, Beaconsfield, UK) according to the method of Lichtenthaler et al. [46]. The fluorescence intensity in the range of blue-green light (430–650 nm) was observed with excitation at 390 nm and near and far red (650–800 nm), and with blue excitation at 430 nm. The slit for the excitation radius was 15 nm and for the emitted radius 20 nm. Based on the spectra, the fluorescence intensity indicators were determined: F440/F530, F440/F6950, F440/F735, and F690/F735. The results were analyzed using the FL WinLab version 3.00. The activity of the cortical (C) and antenna (A) parts of the PSI and PSII systems was determined based on Jena et al. [47]. Fluorescence emission coefficients were measured for leaves from 5 individuals per habitat.

### 2.7. Hydrogen Peroxide Content

The DAB method (3,3′-diaminobenzidine—DAB staining), developed by Daudi, O’Brien [48], was used to determine the content of hydrogen peroxide. DAB is oxidized by hydrogen peroxide in the presence of some heme-containing proteins, such as peroxidases, to generate a dark brown precipitate. This precipitate is exploited as a stain to detect the presence and distribution of hydrogen peroxide in plant cells [49]. The presence of more hydrogen peroxide in different types of plant tissue indicates stressful conditions [50]. The content of hydrogen peroxidase was analyzed for leaves from 5 individuals per habitat.

### 2.8. Electrolyte Leakage

The percentage of electrolyte leakage was carried out following the method used in the study by Możdżeń et al. [51]. *F. vulgaris* leaves (from 5 individuals per habitat) were placed in polypropylene falcons with 30 mL of distilled water, conductivity 0.05 µS. Each falcon was shaken for 3 h on a shaker (Labnet, Rocker, New York, NY, USA) to determine electrolyte leakage from viable leaves (L1). To macerate the material, the leaves were frozen in distilled water at −75 °C for 24 h. The next day, the samples were thawed and subjected to the same procedures described above, and the amount of electrolyte leakage from the dead leaves (L2) was determined. Analyses of the degree of destabilization of cell membranes were measured using a conductometer CX-701 (Elmetron, Zabrze, Poland) with an electrode with a constant K = 1.02 (Elmetron, Zabrze, Poland). Based on the obtained values of L1 and L2, the percentage electrolyte leakage (EL) was determined according to the following formula:

EL = (L1/L2) × 100

EL—a percentage of electrolyte leakage, L1—electrolyte leakage in living cells, and L2—a percentage of electrolyte leakage from dead cells.

### 2.9. Plant Biomass

Plants collected in the field were wrapped in filter paper and transported in a thermo-insulating bag to the laboratory. Plants (10 individuals per habitat) were divided into underground and aboveground parts and their fresh weight (FM) was determined on a laboratory balance (Ohaus Adventurer Pro, Parsippany, NJ, USA). Subsequently, plant organs were dried in an incubator SUP-100 (Wamed, Zabrze, Poland) at 105 °C in order to determine the dry mass (DM). Based on the obtained mass values, the water content in the examined plant organs was calculated:

H_2_O (%) = 100 − [(DM × 100)/FM]

H_2_O—water, DM—dry mass, and FM—fresh mass.

### 2.10. Statistical Analyses

The results were obtained from 5 (chlorophyll content, chlorophyll a fluorescence, fluorescence emission coefficients, mycorrhizal samples) and 10 (electrolyte leakage, plant biomass) repetitions for each of the tested objects. A non-parametric Mann–Whitney U test was performed to test the significance of differences in soil properties between two habitat types. Tukey’s test (for unequal sample size) was used to test the statistical differences for physiological parameters. The analyses were performed using Statistica 13.0 (StatSoft, Tulsa, OK, USA).

## 3. Results

### 3.1. Soils

Soil analysis in terms of the content of macronutrients (Ca, P, K, and Mg) showed that soils from xerothermic grasslands (XG) contained significantly more of these components than soils from *Molinia* meadows (MM)—in soils with grasslands, only in the case of iron (Fe) was lower content found. On the other hand, the soils from the xerothermic grasslands contained significantly fewer heavy metals (Cu, Zn, Cd, and Pb) than the samples of soils from the *Molinia* meadows. In the case of pH, higher values of this parameter were observed on soils from xerothermic grasslands (Table 1).

### 3.2. Degree of Arbuscular Mycorrhizal Fungi (AMF) Colonization of Roots and Morphology of Arbuscular Mycorrhiza (AM)

**Figure 1:**
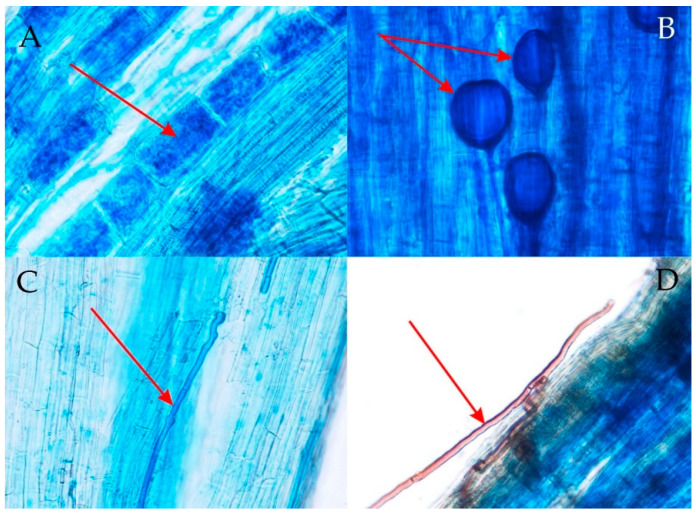
Fragments of the root system of *Filipendula vulgaris* Moench. Arbuscules inside cortical cells (**A**) and vesicles formed by arbuscular mycorrhizal fungi (AMF) (**B**); mycelia of dark septate endophytes (DSE) (**C**,**D**) (photo: M. Fecowicz).

The presence of AM was observed in the roots of all analyzed stands. Parameters of AMF colonization degree oscillated around high values, namely, F—between 97% and 100%, M—between 84% and 93%, and A—between 59% and 81% amongst particular stands. *F. vulgaris* formed *Arum* type of AM (Figure 1). The presence of DSE was found only in two stands: in the succession phase of the xerothermic grassland (F—33%) and the *Molinia* meadow (F—20%). The presence of spores of *Olpidium* spp. has not been recorded.

### 3.3. Physiological Parameters

**Figure 2:**
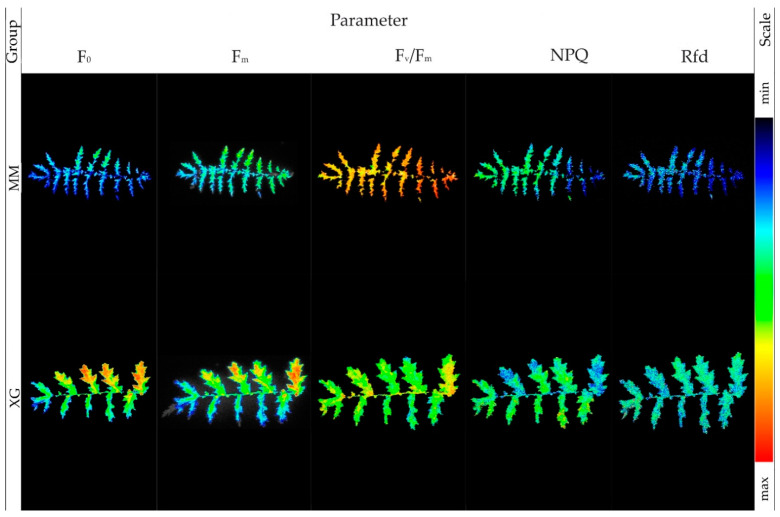
Imaging of chlorophyll *a* fluorescence of leaves of *Filipendula vulgaris* Moench specimens collected from *Molinia* meadows (MM) and xerothermic grasslands (XG).

Imaging of zero and maximum fluorescence showed a lower photosynthetic activity of the dropwort plants from the *Molinia* meadows compared to the plants from xerothermic grasslands (Figure 2).

The activity of PSII measured by the F_v_/F_m_ parameter was higher in the plants from the *Molinia* meadows than in the xerothermic grasslands. In the case of plants from *Molinia* meadows, the NPQ values were lower in the lower part of the leaves than in the upper part. In plants from xerothermic grasslands, NPQ reached similar values on the entire leaf surface. Additionally, these values were higher in relation to plants from meadows. Values of Rfd in the whole surface of leaves were lower in plants from *Molinia* meadows than in xerothermic grasslands.

**Figure 3:**
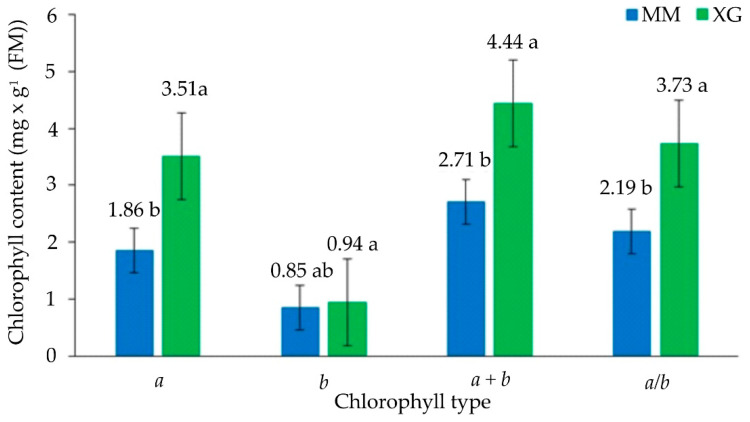
Chlorophyll content in the leaves of *Filipendula vulgaris* Moench collected from the *Molinia* meadows (MM) and xerothermic grasslands (XG); *a*—content of chlorophyll *a*, *b*—content of chlorophyll *b*, total chlorophyll—*a* + *b*, ratio chlorophyll *a* to *b*—*a*/*b*; mean values (n = 5, ±SD) marked with different letters differ significantly according to Tukey’s test (for different N) at *p* ≤ 0.05.

The content of chlorophyll *a* was significantly higher in plants from xerothermic grasslands in comparison with plants from *Molinia* meadows (Figure 3). In the case of chlorophyll *b*, the obtained values did not differ statistically between stands. However, a lower content of this dye was observed in plants from meadows. The sum of chlorophylls (*a* + *b*) was significantly higher in plants from grasslands than in plants from meadows; similarly, the values of the ratio of chlorophylls *a* to *b*.

**Figure 4:**
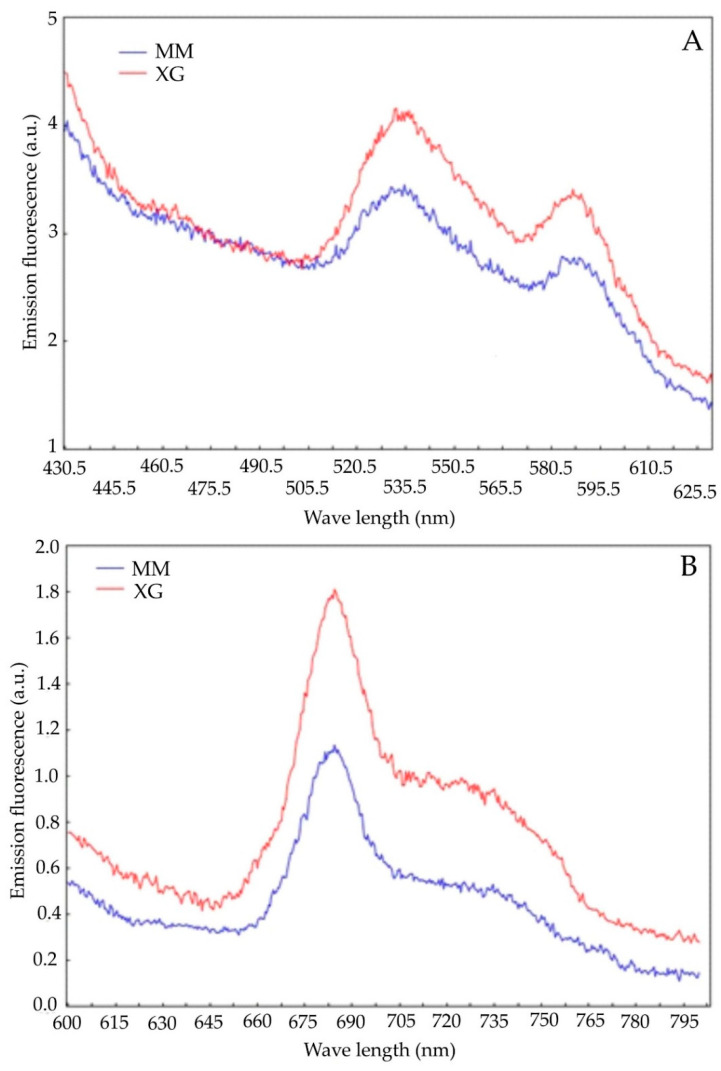
The emission spectra of blue–green (**A**) and red (**B**) fluorescence of *Filipendula vulgaris* Moench leaves collected from the areas of the *Molinia* meadows (MM) and xerothermic grasslands (XG).

The shape of the blue–green and red fluorescence spectra in *F. vulgaris* from the *Molinia* meadows (MM) and xerothermic grasslands (XG) was similar (Figure 4A,B). The blue–green fluorescence spectra showed a slight peak at approximately 535 nm and a second peak at 590 nm (Figure 4A). In the case of red fluorescence, a strong peak of fluorescence was observed at approximately 690 nm, with a very distinct shoulder at 735 nm (Figure 4B). The fluorescence intensity was higher in the plants from the areas of xerothermic grasslands (XG) than in the *Molinia* meadows (MM). Significant differences in the values of the fluorescence emission coefficients were demonstrated between *F. vulgaris* plants from meadows and grasslands (Table 2).

Lower values of fluorescence emission coefficients were recorded for plants from grasslands (XG) than for meadows (MM) and they were statistically significant. The differences in the F690/F35 coefficient were insignificant, although the value was also higher in the case of meadows; similarly, no significant statistical differences were found for the PSI and PSII coefficients.

**Figure 5:**
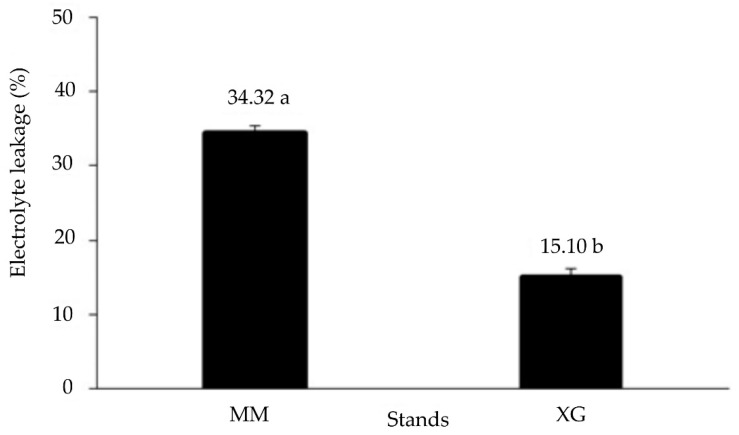
The leakage of electrolytes from the leaves of *Filipendula vulgaris* Moench collected from the areas of *Molinia* meadows (MM) and xerothermic grasslands (XG); mean values (n = 10, ±SD) marked with different letters a, b differ significantly according to Tukey’s test (for different N) at *p* ≤ 0.05.

The percentage of electrolyte leakage from *F. vulgaris* leaf cells was higher by half in plants from the areas of *Molinia* meadows than in xerothermic grasslands (Figure 5).

**Figure 6:**
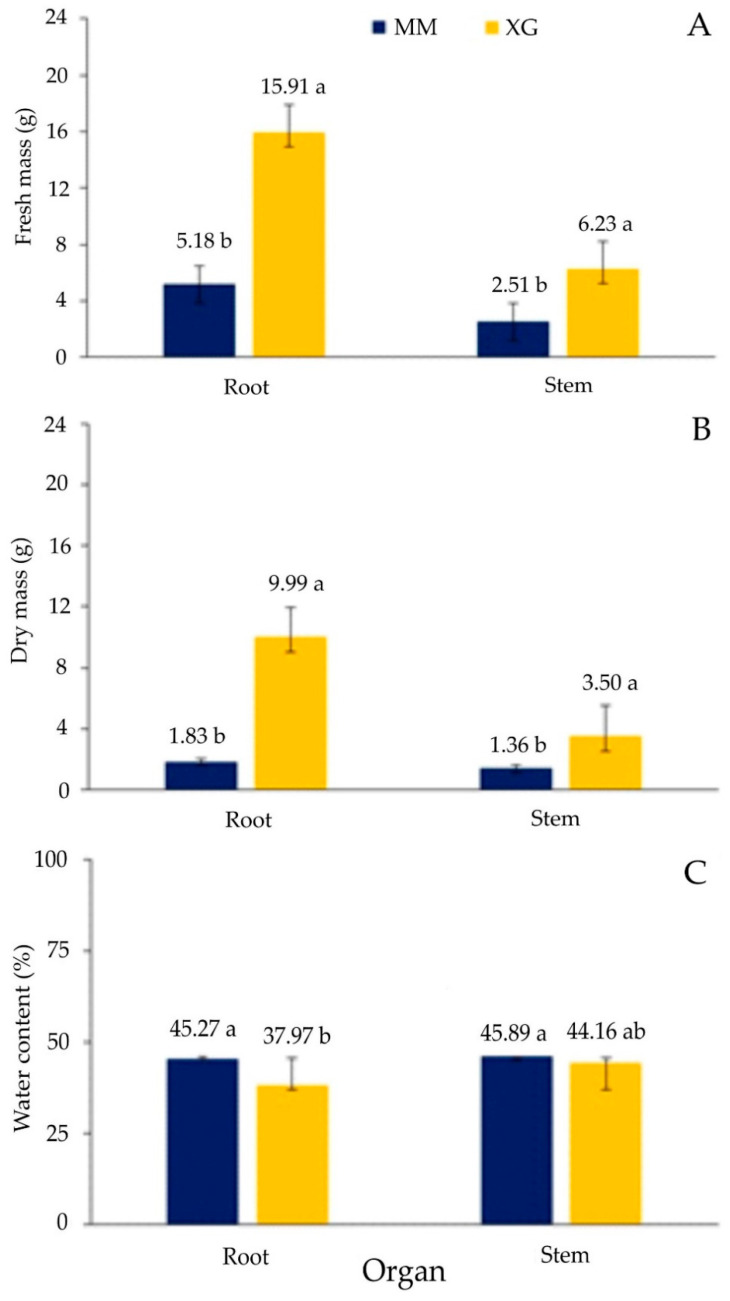
Values of fresh (**A**) and dry mass (**B**) and water content (**C**), organs of *Filipendula vulgaris* Moench plants taken from the areas of *Molinia* meadows (MM) and xerothermic grasslands (XG); mean values (±SD) marked with different letters a, b differ significantly according to Tukey’s test (for different N) at *p* <0.05.

In the case of fresh and dry root mass of *F. vulgaris*, significantly higher values were observed in plants from xerothermic grasslands than in the *Molinia* meadows (Figure 6A–C).

The water content in underground organs was lower in grassland plants than in meadows (Figure 6C). Mass values for dropwort shoots were higher in plants growing on grasslands than in meadows (Figure 6A,B). The percentage of dry mass and water content did not differ statistically between the plants in the studied areas (Figure 6A–C).

**Figure 7:**
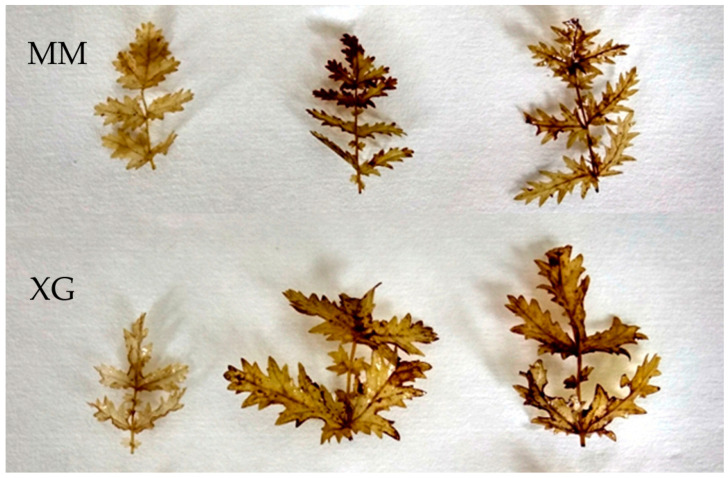
Occurrence of hydrogen peroxide (dark, brown color) in the stem leaves of *Filipendula vulgaris* Moench collected from the areas of *Molinia* meadows (MM) and xerothermic grasslands (XG) (photo: K. Możdżeń).

In the experiment carried out, higher production of hydrogen peroxide was noted in *F. vulgaris* plants collected from the *Molinia* meadows, compared to the specimens from xerothermic grasslands. This was seen as more of the dark brown precipitate (leaf spots) resulting from the oxidation of 3,3′-diaminobenzidine by hydrogen peroxide (Figure 7).

## 4. Discussion

The natural content of chemical elements in the soil depends on the mineralogical composition of the parent rock and the geogenic and pedogenic processes that shape the structure and properties of the soil profile. Soil participates in the circulation of biogenic elements (C, N, P, K, Ca, Mg, Na, and S); the processes of decomposition and synthesis of mineral and organic compounds take place there, as well as their movement and accumulation in the soil profile [52,53,54,55]. The collected results showed a higher content of macronutrients and a lower content of heavy metals in soils from xerothermic grasslands as compared to samples from wet *Molinia* meadows (Table 1). This is the basis for the conclusion that soils from xerothermic grasslands create potentially better conditions for the growth and development of dropwort. The better condition of the dropwort population from xerothermic grassland than wet meadow was also concluded in the previous research of Kostrakiewicz-Gierałt and Stachurska-Swakoń [27], where the population from xerothermic grasslands was more numerous, with better parameters according to seedling recruitment and specimen size. The lower content of heavy metals is important already during the germination of plants, and the phytotoxicity of these elements increases with their concentration in the soil [56,57,58]. Germination is among the first steps in the contact of seeds with a stress factor, which makes them a specific indicator of sensitivity and a measure of tolerance to chemical and physical conditions of the rhizosphere [59,60,61,62].

Environmental stress leads to anatomical, morphological, and physiological changes, including in root tissues, causing inhibition of the water, and ion transport function. The specific surface area of the roots depends on the number and size of intercellular spaces and the properties of the cell walls [63]. An additional factor that often affects plants is microorganisms inhabiting the roots. *F. vulgaris* was included in the species that interact with fungi in the form of arbuscular mycorrhiza (AM) [64] (Figure 1) and showed high values of the mycorrhizal frequency (F). This observation is consistent with the present study, in which the parameter F fluctuated around high values. Previous experiments have shown that the presence of AM increases resistance to abiotic and biotic stresses affecting plants [64,65,66] and affects the proper functioning of plants [67]. In addition, the presence of AM shows a positive effect on the soil structure [68,69,70].

The basic condition for the tolerance of plants to changes in the parameters of the habitat is the quick reception of signals from the external environment and the plants taking up the so-called adaptation decisions, consisting in launching or modernizing programs, conditioning the life processes course. This includes the coordination between the production of nutrients and their distribution throughout the organism. Stress imposes the need to start energy-consuming processes related to acclimatization and adaptation, and limits photosynthetic production, e.g., [71,72]. The efficiency of the photosynthesis process can therefore be an excellent indicator of the condition of plants in specific habitat conditions [73,74,75]. In the experiment carried out here, in specimens of *F. vulgaris* from grasslands, high values of F_0_ indicate a lower efficiency of transferring the excitation energy between chlorophyll molecules [76,77], compared to specimens from meadows (Figure 2). The maximum photochemical efficiency of PSII, expressed as F_v_/F_m_, was higher in plants from *Molinia* meadows, which in grassland specimens indicates a lower potential photochemical efficiency in PSII [78,79]. Drożak and Romanowska [80] showed that the decrease in the F_v_/F_m_ value of *Zea mays* L. may be a consequence of high light intensity. When the light intensity is too high, some of the energy cannot be absorbed by the photosynthetic pigments. This leads to a dysfunction of the photosynthetic structures [81]. In conditions of high radiation, there is an excessive influx of photons to the antennas, which causes an excess of excitation states in the PSII reaction centers [82]. This may partly explain the result obtained in these studies. However, the imaging of F_m_—the maximum fluorescence of chlorophyll a, after a reduction in acceptors in PSII, showed lower photosynthetic activity of *F. vulgaris* specimens from *Molinia* meadows compared to plants from grasslands (Figure 2). Lower values of this parameter may indicate the occurrence of environmental stress other than on grasslands, to which the specimens from meadows are subjected [83,84], e.g., seasonal changes in soil moisture. A consequence of this stress may be that not all electron acceptors in PSII are completely reduced [76]. Parameters such as non-photochemical quenching (NPQ), i.e., dissipation of excess energy in the form of heat, and the PSII vitality index (Rfd), achieved higher values in plants from xerothermic grasslands. In the above-mentioned experiment with maize, the lowest NPQ values were recorded at a low intensity of photosynthetically active radiation [80]. According to some researchers, the increase in NPQ may be the effect of increased thermal energy dissipation [85], which would not be a strange phenomenon in xerothermic grasslands.

Costa et al. [86] observed that the mechanisms to dissipate excess absorbed energy as the heat did not sufficiently prevent photoinhibition. Too high light intensity reduces the content of chlorophyll in plants, while low light intensity increases the content of this pigment. The spectral composition also plays an important role in this regulation; blue light tends to lower the relative chlorophyll content of cells, while red light has the opposite effect [87]. In the specimens of *F. vulgaris* from *Molinia* meadows, the content of chlorophyll *a* (also other values of parameters related to dyes) was significantly lower than in xerothermic grasslands (Figure 3). This pigment absorbs most of the energy from violet–blue and orange–red light wavelengths and is the primary electron donor in the electron transport chain; thanks to it, solar energy is finally converted into chemical energy [88]. Therefore, its higher content directly affects the efficiency of the photosynthesis process. Perhaps the fluorescence of chlorophyll, as well as the mechanism of energy dissipation in the form of heat, plays a very important role here in removing excess light energy absorbed [89].

Changes in fluorescence emission or its proportion (e.g., blue/red) can be indicators of plant stress or an estimate of chlorophyll content [90]. The blue–green fluorescence (F450–F530) results from the presence of phenolic compounds in the leaves’ epidermis, e.g., [91,92]. Under the influence of stress, the plant produces phenolic compounds, which is probably a sign of stress resistance development [93]. In the research with *F. vulgaris* in the field of blue–green fluorescence, two very distinct bands can be distinguished at wavelengths of 535 and 590 nm, both for specimens from meadows (MM) and xerothermic grasslands (XG) (Figure 4A). In the case of fluorescence in the red range, the maximum emission was observed between 685 and 690 nm (most often F690) and in the far-red range near 735 nm (F735) (Figure 4B). The source of fluorescence emission in this range is chlorophyll, which is the PSII reaction center and antenna complexes [94]. Higher values of fluorescence emissions are probably related to the higher content of chlorophyll recorded in the case of grassland specimens (Figure 4). On the other hand, higher values of the fluorescence emission factors indicate greater environmental stress, in this case in the meadow areas (Table 2). This is additionally confirmed by the factor 690/735, which is inversely proportional to the chlorophyll content [95,96]; its higher value means less chlorophyll in plants from *Molinia* meadows (MM). Dropwort specimens from *Molinia* meadows are also characterized by a greater leakage of electrolytes from the leaf cell membranes (Figure 5) and greater production of hydrogen peroxide (Figure 7), which is also a significant sign of environmental stress, e.g., [4,57,97].

Drought is among the many factors that limit the occurrence of plants in the environment. Weather conditions such as low air humidity, high temperatures, and strong winds make rainfall less effective, resulting in drought [98]. Mishra et al. [99] found that under the influence of prolonged drought stress, the stomata close, and CO_2_ assimilation is inhibited. In xerothermic grasslands, drought stress is a permanent element of the habitat. Only after the spring thaw and rainfall, the water conditions are good here. However, as the growing season progresses, the drought stress on xerothermic grasslands increases drastically. For some plants, including *F. vulgaris*, this stress is not that severe, which can be illustrated by the results of the analysis of the fresh and dry mass of individuals obtained here (Figure 6). Although in leaf tissues the percentage of water content is higher in specimens from *Molinia* meadows, both fresh and dry leaf mass are significantly greater in plants from xerothermic grasslands. Probably, better soil conditions may play a more important role than drought in obtaining greater parameters of the mass of individuals on grasslands. The soils of xerothermic grasslands are rendzinas rich in macronutrients, including calcium. For example, it has been established that calcium (Ca^2+^) is an essential macronutrient and plays an important role in plant tolerance to environmental stresses [100,101,102]. The role of Ca^2+^ in relieving drought stress has also been studied in various plants such as *Arabidopsis thaliana* (L.) Heynh. and maize [103,104]. The reason for the lower biomass of plants from the meadows may also be the excess water that occurs periodically in the *Molinia* meadows [105]. These phenomena certainly require further experiments.

## 5. Conclusions

(1) Soil analysis showed a higher content of nutrients in xerothermic grasslands, and a higher content of heavy metals in wet *Molinia* meadows. (2) Plants growing in the wet *Molinia* meadows achieved a significantly lower mass compared to plants growing in the areas of xerothermic grasslands; AMF colonization degree of *Filipendula vulgaris* from both stands oscillated around high values. DSE presence was sporadic and their mycelia were present only in two stands, from stages of xerothermic grassland and *Molinia* meadow. (3) A much higher content of chlorophylls was observed in plants from xerothermic grasslands. (4) *F. vulgaris* showed different photosynthetic activity depending on the habitat; based on chlorophyll *a* fluorescence imaging, higher activity was found in plants from grasslands, compared to plants from *Molinia* meadows, but in specimens from grasslands there are symptoms of damage to the PSII system; however, higher values of fluorescence emission factors indicate greater environmental stress in the meadows. (5) It is also confirmed by significantly higher values of destabilization of cell membranes in leaves of *F. vulgaris* in plants collected from *Molinia* meadows and a higher production of hydrogen peroxide (6).

Despite the wide range of *F. vulgaris* occurrence, the conducted laboratory analyses have shown that in most of the parameters studied (Table 3), better conditions for the growth and physiological activity of this species are probably associated with xerothermic grasslands on a calcareous substrate, although the influence of light stress is also visible here.

## Figures and Tables

**Table 1:** The content of available macroelements and heavy metals as well as the pH in soil samples from the *Molinia* meadows (MM) and xerothermic grasslands (XG); mean values (n = 5, ±SD) marked with different letters differ significantly according to a Mann–Whitney test at *p* ≤ 0.05.

Stands	pH (in KCl)	(mg/kg)										
		Ca	P	K	Mn	Mg	Fe	Cu	Zn	Cd	Pb	NO_3_-N
**MM**	5.05 b	5218.32 b	42.26 b	86.97 b	16.75 b	179.10 b	354.04 a	3.51 a	97.33 a	0.96 a	12.45 a	8.19 a
**XG**	6.67 a	6644.33 a	178.94 a	281.27 a	186.84 a	217.21 a	77.28 b	2.50 b	59.35 b	0.85 b	11.74 b	7.73 a

**Table 2:** Fluorescence emission coefficients of *Filipendula vulgaris* Moench leaves collected from the areas of *Molinia* meadows (MM) and xerothermic grasslands (XG); mean values (n = 5, ±SD) marked with different letters a, b differ significantly according to Tukey’s test (for different N) at *p* ≤ 0.05.

Stands	F440/F530	F440/F690	F440/F735	F690/F735	PSI	PSII	PSI/PSII
**MM**	1.06 a ±0.04	4.14 a ±0.76	7.69 a ±0.56	1.91 a ±0.17	1.48 a ±0.11	1.07 a ±0.14	1.41 a ±0.26
**XG**	0.95 b ±0.04	2.41 b ±0.28	4.02 b ±0.39	1.68 a ±0.18	1.31 ab ±0.06	1.05 a ±0.11	1.31 ab ±0.12

**Table 3:** Comparison of the studied parameters concerning the habitat and physiology of *Filipendula vulgaris* L.; the results of the assessment of the studied habitats and the physiological condition were differentiated by colors: favorable for the plant (green), unfavorable (red) or neutral (blue).

Successive No.	Parameter	*Molinia* Meadows	Xerothermic Grasslands
Soils			
1.	pH in KCl		
2.	macronutrients		
3.	heavy metals		
Parameters of AMF colonization			
4.	F—mycorrhizal frequency		
5.	M—relative mycorrhizal root length		
6.	A—relative arbuscular richness		
7.	DSE presence		
Chlorophyll *a* fluorescence			
8.	F_0_—zero fluorescence		
9.	F_m_—maximum fluorescence		
10.	F_v_/F_m_—maximum photochemical efficiency of PSII		
11.	NPQ—non-photochemical quenching		
12.	Rfd—PSII vitality indicator		
Chlorophyll content			
13.	Chl *a*		
14.	Chl *b*		
15.	Sum *a* + *b*		
16.	Ratio a/b		
Chlorophyll fluorescence emission			
17.	F440/F530		
18.	F440/F6950		
19.	F440/F735		
20.	F690/F735		
21.	PSI		
22.	PSII		
23.	PSI/PSII		
Others			
24.	Hydrogen peroxide content		
25.	Electrolyte leakage		
26.	Fresh mass		
27.	Dry mass		
28.	Water content		

## Data Availability

All additional materials and data are available from the authors.
